# Physiological and Molecular Investigation of Urea Uptake Dynamics in *Cucumis sativus* L. Plants Fertilized With Urea-Doped Amorphous Calcium Phosphate Nanoparticles

**DOI:** 10.3389/fpls.2021.745581

**Published:** 2021-12-07

**Authors:** Sebastian B. Feil, Giacomo Rodegher, Federica Gaiotti, Monica Yorlady Alzate Zuluaga, Francisco J. Carmona, Norberto Masciocchi, Stefano Cesco, Youry Pii

**Affiliations:** ^1^Faculty of Science and Technology, Free University of Bozen-Bolzano, Bolzano, Italy; ^2^Council for Agricultural Research and Economics-Research Centre for Viticulture and Enology, Conegliano, Italy; ^3^Dipartimento di Scienza e Alta Tecnologia and To.Sca.Lab, University of Insubria, Varese, Italy

**Keywords:** cucumber, gene expression, hydroxyapatite, ionomics, nanofertilizers, urea uptake rate

## Abstract

At present, the quest for innovative and sustainable fertilization approaches aiming to improve agricultural productivity represents one of the major challenges for research. In this context, nanoparticle-based fertilizers can indeed offer an interesting alternative with respect to traditional bulk fertilizers. Several pieces of evidence have already addressed the effectiveness of amorphous calcium phosphate-based nanoparticles as carriers for macronutrients, such as nitrogen (N), demonstrating increase in crop productivity and improvement in quality. Nevertheless, despite N being a fundamental nutrient for crop growth and productivity, very little research has been carried out to understand the physiological and molecular mechanisms underpinning N-based fertilizers supplied to plants *via* nanocarriers. For these reasons, this study aimed to investigate the responses of *Cucumis sativus* L. to amorphous calcium phosphate nanoparticles doped with urea (U-ACP). Urea uptake dynamics at root level have been investigated by monitoring both the urea acquisition rates and the modulation of urea transporter *CsDUR3*, whereas growth parameters, the accumulation of N in both root and shoots, and the general ionomic profile of both tissues have been determined to assess the potentiality of U-ACP as innovative fertilizers. The slow release of urea from nanoparticles and/or their chemical composition contributed to the upregulation of the urea uptake system for a longer period (up to 24 h after treatment) as compared to plants treated with bulk urea. This prolonged activation was mirrored by a higher accumulation of N in nanoparticle-treated plants (approximately threefold increase in the shoot of NP-treated plants compared to controls), even when the concentration of urea conveyed through nanoparticles was halved. In addition, besides impacting N nutrition, U-ACP also enhanced Ca and P concentration in cucumber tissues, thus having possible effects on plant growth and yield, and on the nutritional value of agricultural products.

## Introduction

The population inhabiting the Earth is predicted to reach almost 10 billion by 2050 (Taiz, 2013). The agriculture sector is, therefore, asked to increase the production of staple foods in order to satisfy the request of the growing population (Tilman et al., 2011; Kagan, 2016). However, due to soil degradation, the enhancement of agricultural yields could be necessarily obtained by intensifying production rather than extending cultivated areas (Lal, 2015; Kopittke et al., 2019). Although in the past decades the yield increase of key crops has been achieved by raising the use of agrochemicals (i.e., fertilizers and pesticides/herbicides), such massive applications, especially of N-based fertilizers, have posed severe environmental risks (Tilman et al., 2002). Indeed, the effectiveness of conventional fertilizers is threatened by their limited nutrient use efficiency (NUE), i.e., the ability of plants to acquire nutrients at root level and then to allocate them to the shoot and other sink organs. In general, low NUE values originate from fertilization rates that are higher than the ability of plants to take up nutrients (Guo et al., 2018). At present, for instance, it has been estimated that the average NUE for N-based fertilizers is approximately 50%, thus implying that half of the total N applied is not used by crops and lost in the environment (Lassaletta et al., 2014). To overcome these issues, several solutions have been adopted, for instance, soilless cultivation (Sambo et al., 2019). However, the development of new types of fertilizers might also represent a suitable approach to help intensify the agriculture production in a sustainable manner. In this context, the exploitation of nanotechnologies for the delivery of nutrients to crops can be envisaged as an interesting novel solution (Kopittke et al., 2019).

Nanotechnology offers the possibility of creating nanoparticles characterized by smaller size (typically below 100 nm) with respect to bulk materials, and by both high surface area and high reactivity (Ullah et al., 2020). These characteristics open a wide range of opportunities for the development of nanofertilizers featuring higher effectivity, lower ecological risks, and lower economics costs, as compared to their traditional counterparts (Ullah et al., 2020). Interestingly, the use of nanomaterials as carriers will allow tuning the release of the fertilizers in a controlled manner. Such slow release will indeed help prolonging the nutrient persistence in the agro-ecosystem and providing crops with optimal nutrients levels for a prolonged period of time, thus resulting in higher NUE (Guo et al., 2018). According to Kopittke et al. (2019), a wide range of nanomaterials can serve as fertilizers in agriculture, thereby including those composed of the nutrient to be delivered as well as those loaded with the nutrient(s) of interest. In this context, a great attention has been given lately to nanocrystals of hydroxyapatite [HA, Ca_5_(PO_4_)_3_OH] (Kopittke et al., 2019). Nanosized hydroxyapatite presents a high surface area to volume ratio and also the potential of releasing macronutrients, namely, calcium (Ca) and phosphorus (P), for fertilization purposes (Guo et al., 2018). According to these characteristics, HA-based nanoparticles have been studied to improve, on one hand, P nutrition in plants (Liu and Lal, 2014; Xiong et al., 2018; Kopittke et al., 2019; Sega et al., 2019) and, on the other hand, as a carrier to deliver other important macronutrients, such as N (Kottegoda et al., 2011, 2017; Madusanka et al., 2017). Nevertheless, in spite of the importance of N as a plant macronutrient and its relevance for crop productivity, very few studies have been published on the topic so far (Kopittke et al., 2019). First evidence gathered on urea-modified HA nanoparticles showed that the nanofertilizer was effective in releasing urea over a period of 60 days, in contrast with the rapid solubilization of the traditionally used bulk urea (Kottegoda et al., 2011), ensuring a 50% increase in the yield of *Oryza sativa* plants, as compared to controls (Kottegoda et al., 2017). More recently, amorphous calcium phosphate (ACP) nanoparticles, have been developed and doped with urea (U-ACP) (Ramírez-Rodríguez et al., 2020a; Carmona et al., 2021). Similarly to urea modified HA, U-ACP showed a controlled release of macronutrients in soil over time and, most importantly, allowed a wheat grain yield comparable to that of control plants, albeit the N dose applied with U-ACP has been halved as compared to controls (Ramírez-Rodríguez et al., 2020a,b). Interestingly, U-ACP also showed to impact the quality of agricultural products. In Tempranillo grapevine plants, the foliar application of a U-ACP suspension, conveying a N fertilization rate reduced by about 15 times as compared to traditional viticulture practice, induced an increase in yeast-assimilable nitrogen (YAN) and in amino acid concentration at berry level, thus demonstrating a positive impact on the fermentability of grapes (Pérez-Álvarez et al., 2021). More recently, a 2-year study carried out on Pinot gris grapevines highlighted that the fertilization practice with U-ACP, at a 20% lower N fertilization rate as compared to traditional practices, did not alter the growth parameters of plants, as well as, the yield and its quality parameters (Gaiotti et al., 2021). In this regard, the aromatic profile analyses of berries collected from U-ACP fertilized plants did not present any significant alterations as compared to those produced by urea-fertilized samples (Gaiotti et al., 2021). This result clearly suggests that U-ACP can be a valid alternative to bulk urea with undoubted advantages from both environmental and economic standpoints. However, despite these pieces of evidence, information about the physiological and molecular aspects underpinning their action is still scarce.

Bulk urea represents the most widely used fertilizer in agriculture because of its economic convenience and high N content (Beier and Kojima, 2021). Plants have evolved quite a complex system to take up urea from the external environment that is based on channels, namely, nodulin 26-like intrinsic proteins (NIPs) and plasma membrane intrinsic proteins (PIPs), and on a specific transporter, degradation of urea (DUR) 3. Tonoplast intrinsic protein (TIP) channels have also been identified. While NIPs, PIPs, and TIPs seem to marginally contribute to urea uptake, and just in very specific conditions, DUR3 is accounted as the main carrier for this N form (Liu et al., 2003; Wang et al., 2012; Zanin et al., 2014). DUR3 transporter is localized in root cells plasma membrane, and it has been shown to act as a high affinity proton-dependent symporter (Liu et al., 2003). The transporter is encoded by the *DUR3* gene (Kojima et al., 2007; Wang et al., 2012; Zanin et al., 2014), whose transcriptional modulation shows down-regulation in response to nitrate and ammonium supply, whereas N starvation and urea fertilization induce an up-regulation of *DUR3* expression in several plant species (Beier and Kojima, 2021). These observations corroborate the existence of the so-called induction phenomenon, already demonstrated for nitrate uptake in plants. To this regard, Zanin et al. (2014) showed that maize plants, starved of N and afterward exposed to urea for 4 h, could take up the nutrient with a different kinetic behavior compared to untreated plants, thereby displaying a higher Km and Vmax. Indeed, such increase indicates a higher capacity of the transport system, yet features lower specificity (Siddiqi et al., 1990).

On these premises, the aim of this study was to investigate the physiological and molecular responses of *Cucumis sativus* L. (used a plant model for crops) to exposure to U-ACP. A traditional N fertilizer (i.e., bulk urea) has been used as control. By adopting a time-course experiment, the urea uptake dynamics at root level have been investigated by monitoring both urea acquisition rates and modulation of *CsDUR3*. In a mid-term experiment, on the other hand, plant growth parameters, the accumulation of N in both root and shoots, as well as the general ionomic profile of both tissues, have been analyzed to highlight possible synergism and antagonism between U-ACP and other mineral nutrients.

## Materials and Methods

### Urea-Doped Amorphous Calcium Phosphate Nanoparticles Preparation and Characterization

The nanosized U-ACP material was prepared following a procedure recently reported (Carmona et al., 2021). Briefly, an aqueous solution (solution A, 75 ml) containing 0.4 M calcium chloride and 0.4 M sodium citrate dihydrate was poured on another solution (solution B, 75 ml) containing 0.2 M of sodium carbonate and 0.24 M of potassium phosphate dibasic. The resulting suspension was heated at 37°C for 5 min. Afterward, the slurry was washed with water (2×300 ml) and separated from the supernatant by centrifugation (10 min, 4,500 rpm, 25°C). Then, an aqueous solution of urea (3 ml, 160 g L^–1^) was mixed with the slurry to dope the nanoparticles. Finally, the material was lyophilized at −55°C and recovered as a dry powder.

Amorphous calcium phosphate (U-ACP) was characterized by X-ray powder diffraction (XRPD), Fourier-transformed infrared spectroscopy (FTIR), elemental analysis (EA), and inductively coupled plasma optical emission spectrometry (ICP-OES) (Supplementary Figure 1). The XRPD data were collected on a Bruker AXS D8 Advance diffractometer by Cu Kα radiation (λ = 1.5418 Å). The 2θ range of the measurements 5–55° with a scanning rate of 1 s per step and step size of 0.02°. FTIR spectra were registered on a Bruker Tensor 27 spectrometer. The samples (2 mg) were dispersed in KBr (100 mg), and the mixture was pressed into a pellet. The FTIR spectra were collected with a spectral resolution of 2 cm^–1^ by accumulating 32 scans in the 4,000–450 cm^–1^ range. The content of nitrogen in the sample was determined by elemental analysis, performed with a Perkin Elmer 2400 series II instrument at the Center of Scientific Instrumentation of the University of Granada (CIC-UGR) [N content = 6.43% ± 0.3 (w/w)]. The content of calcium and phosphate ions in the resulting material was quantified by inductively coupled plasma optical emission spectrometry (ICP-OES, Optima 8300, PerkinElmer, CIC-UGR). *Ca*. 20 mg of U-ACP was decomposed in 2 ml of concentrated nitric acid and diluted with deionized water (100 ml) (Ca/P molar ratio = 1.92 ± 0.02).

### Plant Material and Growing Conditions

Seeds of cucumber plants (*Cucumis sativus* L. cv. Chinese Long) were germinated on filter papers, wetted with 0.5 mM CaSO_4_ solution, in a covered tray. After 5 days, the seedlings were transferred into 1.5-L pots containing an aerated hydroponic solution composed of Ca(NO_3_)_2_ (2 mM), MgSO_4_ (0.5 mM), K_2_SO_4_ (0.7 mM), KCl (0.1 mM), KH_2_PO_4_ (0.1 mM), H_3_BO_3_ (10 μM), MnSO_4_ (0.5 μM), CuSO_4_ (0.2 μM), ZnSO_4_ (0.5 μM), (NH_4_)_6_Mo_7_O_24_ (0.01 μM), and Fe-EDTA (80 μM) (Pii et al., 2016). The solution was replaced regularly every 2 days. After 7 days of growth in a full nutrient medium, Ca(NO_3_)_2_ was removed from the solution in order to achieve a period of nitrogen starvation (Supplementary Figure 2A). The Ca^2+^ concentration was rebalanced adding adequate amounts of CaSO4 in each pot. The N starvation condition was maintained for 7 days.

### Induction Experiments

In a preliminary experiment, the responsiveness of the urea uptake system in cucumber plants was checked (Supplementary Figure 2A). To this aim, after 7 days of N starvation, half of the pots were induced by supplying the nutrient solution with 1 mM CO(NH_2_)_2_. The urea uptake rate was determined at 2, 4, 8, 18, 24, and 48 h after the induction by exposing intact root system to an uptake solution containing ^15^N-labeled urea. In detail, the seedlings were removed from the nutrient solution, washed in 0.5 mM CaSO4, and placed for 7 min in a 200-μM CO(^15^NH_2_)_2_ uptake solution titrated at pH 6 with 1 mM MES-KOH. After the uptake, the roots were washed with 0.5 mM CaSO4 and dried in an oven at 65°C for 3 days. Afterward, the roots were homogenized and analyzed by isotope ratio-mass spectrometry (IRMS). At the same time points (i.e., 2, 4, 8 18, 24, and 48 h after the induction), roots were also sampled, frozen in liquid N_2_, and stored at −70°C for further molecular analyses.

The preparation of plant material for the induction experiment involving U-ACP followed the same procedure as described above (Supplementary Figure 2B). After the period of starvation, the pots were separated into four groups; the first was not treated (Not Induced), the second was induced by using 1 mM CO(NH_2_)_2_ (Urea 1 mM), the third was induced with full-strength U-ACP (hereafter referred to as NP), and the fourth was induced with half-strength U-ACP (hereafter referred to as NP.5×). The amount of NP was estimated to supplement plants with the same concentration of N added *via* 1 mM CO(NH_2_)_2_. The determination of the urea uptake rate was carried out as described above.

### Isotope Analysis

Analyses were carried out with an isotope mass spectrometer (Delta V; Thermo Fisher Scientific, Germany) following total combustion in an elemental analyzer (EA Flash 1112; Thermo Fisher Scientific, Germany), as previously described (Pii et al., 2019).

### Gene Expression Analysis

RNA extractions from roots were performed using a Spectrum™ Plant Total RNA (Sigma-Aldrich) kit. The root samples were homogenized in liquid N_2_, and 100 mg of the root powder was subjected to extraction following the manual of the manufacturer. The total RNA samples were treated with 10 U of DNaseRQ1 (Promega), and cDNA was synthesized with the ImProm-II™ Reverse Transcription System (Promega), following the user manual. Quantitative real time RT-PCR was performed using SsoFast™ EvaGreen^®^ Supermix (Bio-Rad) in the CFX96 Touch Real-TimeDetection System (Bio-Rad), on three independent biological replicates (Pii et al., 2019). The target gene, *CsDUR3* (*Cucsa.322410*, retrieved at)^1^ was amplified using a gene-specific primer (Forward 5′-AGAAGCAGATGTATTTAGAAACT-3′ and Reverse 5′-ACTAGATAGGGTGAACTAACAAT-3′) specifically designed to produce amplicons of 110 bp. The expression levels of target genes were normalized with those of the housekeeping genes ubiquitin elongation protein and elongation factor 1-α tubulin (Pii et al., 2016). The value of relative expression ratio was calculated for treated samples relative to the corresponding untreated sample at the same time point, according to the Pfaffl equation (Pfaffl et al., 2002). Standard error values were calculated according to Pfaffl (2001).

### Mid-Term Fertilization Experiment

Cucumber seedlings were germinated and grown as described previously; after 7 days of N starvation, the cucumber plants were supplied with different N sources (i.e., urea 1 mM, NP, and NP.5×) (Supplementary Figure 2C). The plants were sampled at the beginning of the treatments (time 0 days) and 7 days after the fertilization. For each time point, roots and shoot samples were collected to analyze seedling biomass, total N amount, root morphology, ionomic profile, and SPAD index. Tissues collected for biomass determination, total N amount, and ionomic analyses were dried at 65°C until constant weight and subsequently grinded. The determination of N concentration in the root and shoot samples was carried out by isotope-ratio mass spectrometry (IRMS). The root morphology of the cucumber plants was assessed by scanning the roots with a WinRHIZO™ (WinRhizo software, EPSON1680, WinRHIZO Pro2003b; Regent Instruments Inc., Quebec, Canada) system. Leaf relative chlorophyll content was measured at harvest using a portable Minolta SPAD-502 (Konica-Minolta, Osaka, Japan).

### Ionomic Analysis

Root tissues were oven dried at 65°C until constant weight was reached, ground in liquid N_2_, and acid-digested with concentrated HNO_3_ [65% (v/v); Carlo Erba] using a single reaction chamber (SRC, UltraWAVE; Milestone Inc., Shelton, CT, United States). The concentration of elements was subsequently determined by inductively coupled plasma-optical emission spectroscopy (ICP-OES, Spectro Arcos; Spectro, Germany). Quantifications of the elements were carried out using certified multi-element standards (CPI International).^2^ Tomato leaves (SRM 1573a) and spinach leaves (SRM 1547) were used as an external certified reference material.

### Statistical Analysis

All the datasets were tested for normal distribution by quantitative Shapiro–Wilk test. Depending on the dataset, the significance of differences among means was calculated by either Student’s *t*-test, one-way ANOVA with *post hoc* Tukey HSD, or two-way ANOVA, as specified in figure legends. The significance of the clustering observed in the principal component analysis (PCA) was assessed by PERMANOVA test using 5,000 permutations. The statistical analyses and data visualization were carried out using the R software v.3.6.1 using packages listed in Supplementary Information 1.

## Results

### Urea Uptake Rate

Considering the variability reported in the literature in the response of different plant species to urea treatments (Kojima et al., 2007; Wang et al., 2012; Zanin et al., 2014), a preliminary characterization of urea uptake dynamics in *Cucumissativus* L. was carried out (Supplementary Figure 2A). Data showed that the urea-treated plant displayed a typical induction behavior, reaching a peak in the uptake rate 8 h after treatment (hereafter referred to as HAT) and down-regulating the process afterward (Supplementary Figure 3A). Consistently, the enhanced uptake rate observed at 8 HAT in induced plants was related to an up-regulation of the *CsDUR3* gene as compared to non-induced plants (Supplementary Figure 3B).

**FIGURE 1:**
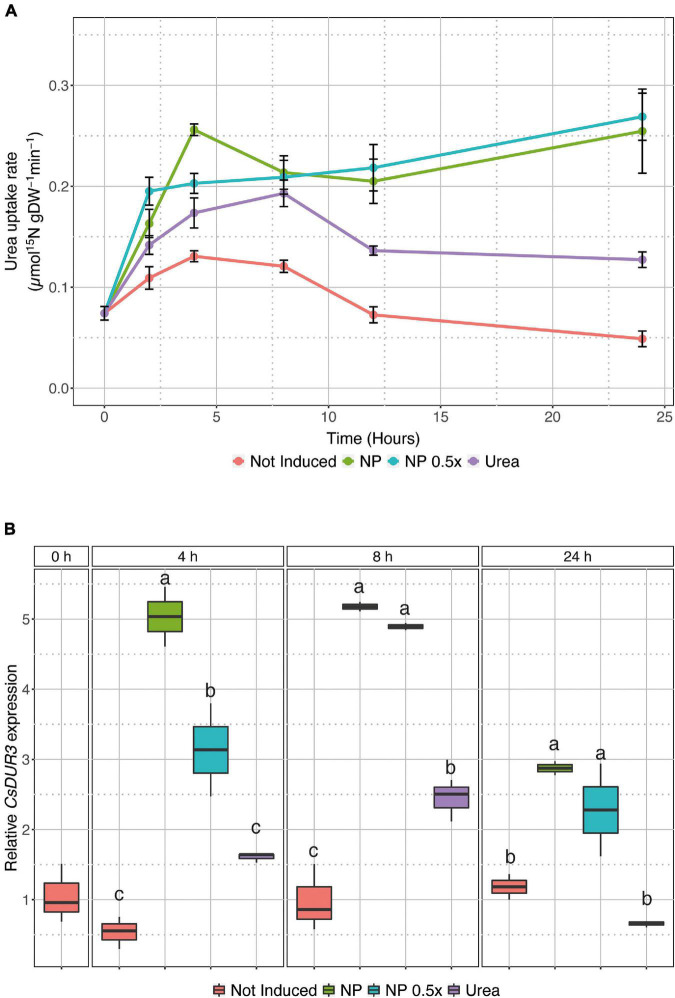
Urea uptake in intact cucumber roots. **(A)** High-affinity urea uptake rate in cucumber roots either not treated (not induced) or treated with 1 mM urea, NP. or NP.5×. Uptake rates were determined by placing the seedlings in 200 μM^15^N-labeled urea solution for 7 min. Data are the means (±SE) of three independent biological replicates; each biological replicate was obtained by pooling five independent plants. The statistical significance of the whole dataset has been tested by two-way analysis of variance (ANOVA) test (Time *p* < 0.05, Treatment *p* < 0.001, Time×Treatment *p* < 0.001), whereas the difference between treatments within each time point was tested by one-way ANOVA with Tukey *post hoc* tests (*P* < 0.001), and the results are reported in Supplementary Table 1. **(B)** Time course expression analysis of *CsDUR3* in cucumber roots either not treated (not induced) or treated with 1 mM Urea, NP, or NP.5×. *CsDUR3* expression levels were assessed by qRT-PCR; data have been normalized to two internal controls, ubiquitin elongation protein, and elongation factor 1-α tubulin. The relative expression ratios were calculated using not-induced roots sampled before the treatments (0 h), which were set with a value of 1. Different letters within a time-point indicate significantly different values as determined by one-way ANOVA with Tukey *post hoc* tests (*P* < 0.001).

To investigate the possible effects of urea-doped amorphous calcium phosphate nanoparticles on the dynamics of N uptake in cucumber plants, U-ACP (full-strength NP and half-strength) was applied in the induction experiments, as depicted in Supplementary Figure 2B. While the not induced and 1 mM urea-induced plants showed a similar pattern of urea uptake rates, although to a different extent (higher in urea-induced ones, Figure 1A and Supplementary Figure 3A), the plants treated with NP behaved differently. Both the NP and NP.5×-treated cucumber plants showed an anticipation in the uptake induction, at 4 and 2 HAT, respectively, as compared to the urea-treated plants, whose induction maxima was reached at 8 HAT (Figure 1A and Supplementary Table 1). Interestingly, in the NP-treated plants, the urea uptake rate did not display a down-regulation within the time interval of the experiment, but it was maintained at the same levels reached soon after the treatments (Figure 1A and Supplementary Table 1). These observations were also further confirmed by *CsDUR3* gene expression dynamics (Figure 1B). In the urea-treated plants, the expression of *CsDUR3* followed the uptake rate profile (Figure 1), resulting in significant upregulation at 8 HAT and no modulation at 24 HAT as compared to the not induced plants (Figure 1B). In the NP-treated plants, *CsDUR3* was strongly up-regulated (fivefold) at both 4 and 8 HAT as compared to the not-induced plants. At 24 HAT, *CsDUR3* expression was slightly down-regulated compared to the previous time points, albeit it remained significantly higher as compared to the not-induced and urea-treated plants (Figure 1B). A similar response was observed in the NP.5×-treated plants. At 4 HAT, *CsDUR3* expression was already significantly induced as compared to both the not-induced and urea-treated plants. *CsDUR3* reached the highest expression at 8 HAT, and it declined afterward, despite being significantly more expressed as compared to the not induced and urea-treated plants (Figure 1B). Except for the results obtained at 4 HAT, the induction levels of *CsDUR3* appeared to be independent of the concentration of the applied NP.

### Mid-Term Fertilization Experiment

#### Growth Parameters

**FIGURE 2:**
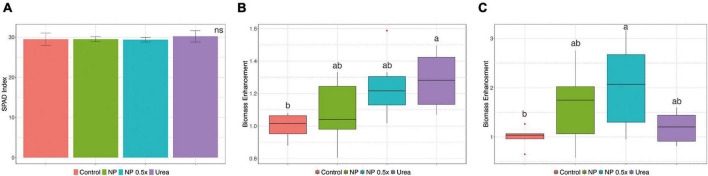
Growth parameters. **(A)** SPAD index, **(B)** shoot, and **(C)** root biomass enhancement of cucumber plants either not treated (control) or supplied with 1 mM Urea, NP, or NP.5×. The assessments have been carried out at harvest (i.e., 7 days after the treatments as depicted in Supplementary Figure 2C) on at least six independent biological replicates. For the SPAD index, data are reported as means ± SE. The biomass enhancement depicts the fold-change variation in the DW biomass of treated cucumber plants as compared to control ones. Different letters indicate significantly different values as determined by one-way ANOVA with Tukey *post hoc* tests (*P* < 0.05).

**FIGURE 3:**
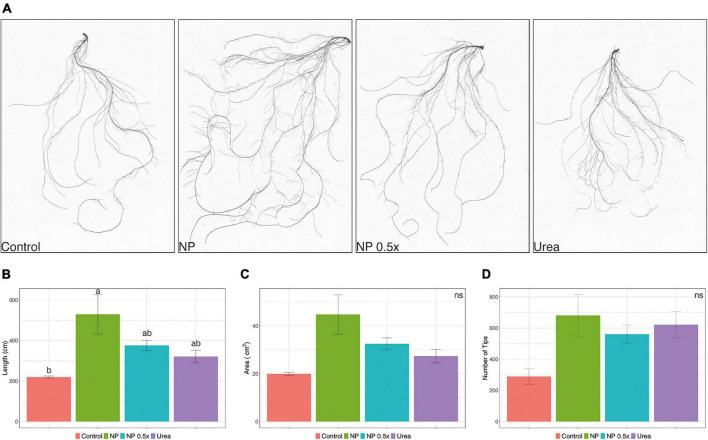
Effects of different treatments on root architecture. **(A)** Representative pictures of cucumber root systems, **(B)** total root length, **(C)** total root surface area, and **(D)** root tips of cucumber plants either not treated (control) or supplied with 1 mM urea, NP, or NP.5×. The assessments have been carried out at harvest (i.e., 7 days after the treatments as depicted in Supplementary Figure 2C) on at least six independent biological replicates. Data are reported as means ± SE. Different letters indicate significantly different values as determined by one-way ANOVA with Tukey *post hoc* tests (*P* < 0.05).

Considering the higher uptake rates observed in the NP- and NP.5×-treated plants, a mid-term fertilization experiment was carried out (Supplementary Figure 2C). At harvest, phenotypic and growth parameters were assessed. The content of chlorophyll in leaves, estimated as SPAD index, did not display significant alterations depending on the fertilization strategy (Figure 2A). Plants fertilized with urea showed a significantly higher accumulation of leaf biomass as compared to control plants, whereas those treated with NP, independently of the concentration, displayed an intermediate biomass value (Figure 2B). At root level, the treatment with NP.5×caused the highest accumulation of biomass as compared to controls, albeit it was not significantly different from the biomass accumulated in plants treated with either NP or urea (Figure 2C). The quantitative assessment of root length parameter (Figures 3A,B) highlighted that the NP-treated plants tended to show the highest extension of the root systems, as suggested by the root pictures (Figure 3A). On the other hand, both urea- and NP.5×-treated plants showed an increasing trend in the root length in comparison with control, despite being not statistically significant (Figure 3B). The analyses of root area (Figure 3C) and number of tips (Figure 3D) showed an increasing tendency with respect to control plants, albeit no statistically significant difference could be highlighted (Figures 3C,D).

#### Accumulation of Mineral Nutrients

**FIGURE 4:**
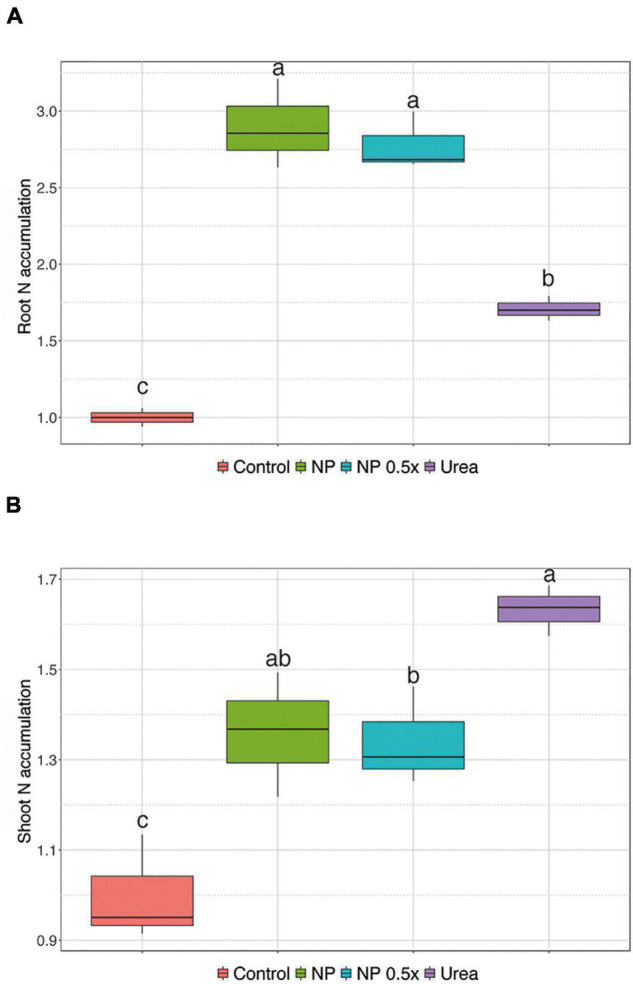
Total nitrogen (N) content. Accumulation of N in **(A)** root and **(B)** shoot of cucumber plants either not treated (control) or supplied with 1 mM Urea, NP, or NP.5×. The assessments have been carried out at harvest (i.e., 7 days after the treatments as depicted in Supplementary Figure 2C) on at least six independent biological replicates. N accumulation has been calculated as the ratio between the concentration assessed in the treated plants and that determined in control samples. Different letters indicate significantly different values as determined by one-way ANOVA with Tukey *post hoc* tests (*P* < 0.05).

To understand whether the increased urea uptake in the first 24 h after the treatments might lead to a higher accumulation of nitrogen (N) in the mid-term experiment, N content was determined in the plants 7 days after the supplementation with different fertilizers. As shown in Figure 4A, the plants treated with 1 mM urea displayed, on average, 1.7-fold increase in N concentration at root level as compared to control plants. Interestingly, the plants treated with NP, regardless of the concentration level, showed the highest values, ranging between 2.5- and 3-fold, of N accumulation in the root tissue with respect to controls (Figure 4A). Despite such prominent accumulation in roots, the highest N concentration was detected in the shoots of urea-fed plants (Figure 4B). The plants treated with NP showed an intermediate level of N accumulation as compared to the urea-fertilized samples, albeit the difference was not significant. On the other hand, the NP.5×treatment caused a lower accumulation of N in cucumber shoots, which was significant when compared to the urea-treated samples and not significant with respect to the NP-treated plants (Figure 4B). Collectively, all the treated plants showed a significant increase in N levels, ranging from 1.3- to 1.7-fold, as compared to the control ones (Figure 4B).

**FIGURE 5:**
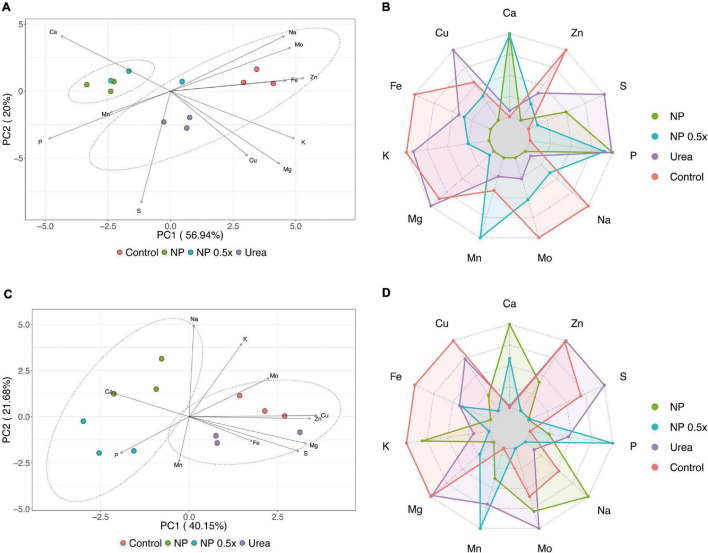
Ionomic analysis. **(A)** Scatter plot reporting the results of the PCA carried out on root ionomic profile of cucumber plants either not treated (control) or supplied with 1 mM urea, NP, or NP.5×. The assessments have been carried out at harvest (i.e., 7 days after the treatments as depicted in Supplementary Figure 2C) on three independent biological replicates. The statistical significance of the difference between the two clusters drawn has been assessed by PERMANOVA analysis with 5,000 permutations (*p* < 0.001). **(B)** Radar chart reporting the variations in element concentration in roots of cucumber plants supplied with 1 mM Urea, NP, or NP.5×with respect to control plants. **(C)** Scatter plot reporting the results of the PCA carried out on shoot ionomic profile of cucumber plants either not treated (control) or supplied with 1 mM Urea, NP, or NP.5×. The assessments have been carried out at harvest (i.e., 7 days after the treatments as depicted in Supplementary Figure 2C) on three independent biological replicates. The statistical significance of the difference between the two clusters drawn has been assessed by PERMANOVA analysis with 5,000 permutations (*p* < 0.001). **(D)**. Radar chart reporting the variations in element concentration in the shoot of cucumber plants supplied with 1 mM Urea, NP, or NP.5×with respect to control plants.

Concerning the concentration of other mineral nutrients, the dataset representing the root ionomic signature was subjected to PCA, which allowed to obtain a four-component model, globally explaining 92.82% of the total variance. The scatterplot obtained by combining principal component 1 (PC1) and PC2 accounted for 76.94% of the total variance and clearly showed the separation of the samples along PC1 in two distinct clusters (PERMANOVA, *p* < 0.001), encompassing the first NP- and NP.5×-treated plants and the second control and urea-treated plants (Figure 5A). According to the loading vectors displayed in Figure 5A, the separation along the PC1 is mainly due to the concentration of P and Ca in the negative direction and to that of Fe, K, Na, Mg, Zn, and Mo in the positive one. Notably, in the cluster formed by the control and urea-treated plants, a separation of sample along PC2 was observed, and it was mainly driven by the concentration of S and Cu in the negative direction (Figure 5A). Indeed, the NP.5 ×—and NP-treated plants showed common features, for instance, prominent accumulation of Ca and P and slight increase in the concentration of S. On the other hand, the control plants displayed high accumulation of K, Na, Mg, Fe, Mo, and Zn as compared to the other treatments (Figure 5B). The urea-treated plants, instead, displayed features common with both controls, for instance, the accumulation of K and Mg, and with the NP- and NP.5×-treated plants, such as increase in P and S concentrations (Figure 5B).

The PCA carried out on the dataset representing the ionome profile of cucumber shoots allowed to obtain a five-component model, describing 91.6% of the total variance. The scatterplot obtained by combining PC1 and PC2, accounting for 61.83% of the total variance, showed the clustering of samples in two distinct groups (PERMANOVA, *p* < 0.001), as also observed at root level (Figure 5C). One cluster encompassed the control and urea-treated plants, while the second cluster was formed by the NP- and NP.5×-treated plants. The two groups were separated along the PC1 and, this separation was mainly driven by Ca and P in the negative direction and by Cu, Mg, S, and Zn in the positive one (Figure 5C). Consistently, the NP- and NP.5×-treated plants showed an accumulation of Ca and P as compared to controls; on the contrary, Cu, Mg, S, and Zn showed a significantly higher concentration in the controls and urea-treated plants in comparison to the NP-treated ones (Figure 5D).

## Discussion

The quest for more sustainable approaches to meet the challenges of a growing world population is forcing agriculture toward the development of innovative fertilization practices (Tilman et al., 2011; Kagan, 2016). In this regard, an attractive solution might be represented by the application of agrochemicals based on nanomaterials, which can feature higher effectivity, lower ecological risks, and lower costs as compared to their traditional counterparts (Ullah et al., 2020). Previous studies have already demonstrated the suitability of nanoparticles based on amorphous calcium phosphate (ACP) nanoparticles as carriers of macronutrients (e.g., urea). In particular, it has been demonstrated that upon reduction of N fertilization rate, plants supplemented with ACP could mirror the same agronomic performance (i.e., yield and quality) of plants treated with bulk fertilizers (Ramírez-Rodríguez et al., 2020a,b; Carmona et al., 2021; Gaiotti et al., 2021; Pérez-Álvarez et al., 2021). Despite this evidence, the knowledge of plant physiological responses to urea-doped ACP (U-ACP) nanoparticles is still scarce.

The data hereby presented corroborate further that urea itself, as previously demonstrated for the inducible high affinity nitrate transport system (Orsel et al., 2002), can function as a signal for the induction of its own specific transport system in cucumber; indeed, the induced plants showed higher urea uptake rate and significant up-regulation in the expression of the *CsDUR3* gene (Supplementary Figure 3 and Figure 1). To date, the kinetics of urea uptake mechanism in higher plants has only been characterized in *Arabidopsis*, rice, and maize (Kojima et al., 2007; Wang et al., 2012; Zanin et al., 2014). Interestingly, maize plants exposed for 4 h to 1 mM urea showed a twofold increase in substrate uptake rate (Zanin et al., 2014), which is consistent with the kinetics data obtained here in the time-course experiment (Supplementary Figure 3 and Figure 1). After the initial up-regulation, the uptake rate showed a decline that could resemble the de-induction phenomenon described in the case of NO_3_^–^ acquisition at root level (Glass et al., 2001; Nikolic et al., 2012). The kinetics of urea uptake in cucumber plants was also supported by the modulation of the *CsDUR3* gene, which was up-regulated at 8 HAT, as compared to the not induced plants, and then down-regulated at 24 HAT (Figure 1B). These results further expand the knowledge of urea uptake dynamics provided by previous studies on different plant species (Kojima et al., 2007; Wang et al., 2012; Zanin et al., 2014). In fact, they are in good agreement with the observations carried out on rice seedlings, in which *OsDUR3* was induced 3 h after treatment with 1 mM urea (Wang et al., 2012). On the other hand, experiments carried out on *A. thaliana* and maize seedlings highlighted the down-regulation of *DUR3* expression 3 and 6 h after the treatment with 1 mM urea (Kojima et al., 2007; Zanin et al., 2014). These observations might suggest a different control of the urea uptake machinery depending on plant species, as also well described for the transcriptional regulation of ammonium transporters (Goyal and Huffaker, 1986; Mack and Tischner, 1994; Kronzucker et al., 1998; Rawat et al., 1999; Sonoda et al., 2003; Loqué and von Wirén, 2004; Yuan et al., 2007; Pii et al., 2019). When the cucumber plants were treated with NP, regardless of the concentration level, the pattern of urea uptake rates showed, at the beginning, a similar induction trend as observed in the urea-treated ones, whereas it did not display a de-induction phase (Figure 1A), with *CsDUR3* modulated accordingly (Figure 1B). The prolonged activation of the urea uptake machinery in cucumber plants treated with NP suggests the ability of these plants to acquire the nutrient for a longer period of time with respect to conventional treatments; this resulted, for instance, in the higher accumulation of N at root level, when a 7-day fertilization period was considered (Figure 4A). Assuming that urea could function as a signal for the induction of its own uptake system (Beier and Kojima, 2021), the acquisition and molecular data obtained from the present experimental system might be ascribable to the kinetics of urea release from nanoparticles. Indeed, it has been demonstrated that up to 90% of the urea loaded on nanoparticles is released within 1 h by U-ACP dispersed in an aqueous solution (Ramírez-Rodríguez et al., 2020a; Carmona et al., 2021). Notwithstanding, the residual urea is released with a slower kinetic, ranging from 24 h up to 1 week, accordingly with the nanoparticles dissolution kinetics (Ramírez-Rodríguez et al., 2020a; Carmona et al., 2021).

Although the cucumber plants fertilized with NP and NP.5×showed higher N accumulation in the roots as compared to both the control and urea-treated plants, N accumulation was higher in the shoots of plants supplied with bulk urea (Figure 4B). In fact, both the NP- and NP.5×-fertilized plants presented lower N content in shoots, albeit this drop was significant only in the case of the NP.5×treatment in comparison to bulk urea. The differences between bulk urea and U-ACP observed in the translocation and allocation at leaf level could be ascribable to the diverse chemical structure of the nutrient. Consequently, the pathways involved in the uptake, translocation, and assimilation mechanisms might also be different. Bulk urea, for instance, is 100% available when it is administrated to plants. On the other hand, when U-ACP nanoparticles are dispersed in an aqueous solution, a quick release of the majority of urea is observed and it is then followed by slow solubilization (Ramírez-Rodríguez et al., 2020a; Carmona et al., 2021). Moreover, recent evidence has also shown that U-ACP nanoparticles can penetrate the root epidermis and reach the central cylinder *via* an apoplastic movement, which should become symplastic to cross the Casparian strip (Pérez-de-Luque, 2017; Ramírez-Rodríguez et al., 2020b). It is also worth noting that the different uptake dynamics could not only be related to the timing of urea release from the nanoparticles, but they could also be due to the nanoparticles themselves. In fact, analog experiences studying Fe nutrition in tomato demonstrated that, depending on the chelating agent (phytosiderophores vs. citrate vs. water extractable humic substances), plants could take up and allocate the micronutrient with different efficiency, albeit Fe was supplied at the same concentration in the growth substrate (Tomasi et al., 2013). Therefore, considering the peculiarities of U-ACP with respect to bulk urea, it might not be surprising that in over a 7-day fertilization period, slight differences in the N content of shoots were observed among the treatments (Figure 4B). Nevertheless, it is interesting to underline that the cucumber plants treated with NP.5×performed better in terms of N acquisition with respect to both the NP and urea treated ones (Carmona et al., 2021).

As also already observed (Carmona et al., 2021), despite the differential accumulation of N in plant tissues, no significant alteration in the biomass accumulation at root and shoot levels as well as in leaf chlorophyll content has been observed in the cucumber plants according to the fertilization treatments (Figure 2).

Several pieces of evidence have demonstrated that the mineral composition of plant tissues is tightly related with different aspects of (i) the edaphic environment (such as the chemical and physical characteristics of the growth substrate) as well as (ii) agricultural practices (such as fertilization and the application of biostimulants, e.g., plant growth-promoting rhizobacteria). The ionomic profiling of plant tissues subjected to different fertilization practices can, for instance, highlight possible phenomena of synergism and/or antagonism between mineral elements, thus resulting in differential accumulation of essential and/or non-essential mineral nutrients (Nikolic et al., 2007; Tomasi et al., 2009; Pii et al., 2015; Astolfi et al., 2018; Kolega et al., 2020; Scagliola et al., 2021). The treatment of cucumber plants with NP, regardless of the concentration level, caused an increased accumulation of calcium (Ca) and phosphorus (P) at both root and shoot levels (Figure 5). It is, however, noteworthy that, by supplying NP in order to meet plant N nutrition requirements, neither P nor Ca reached critical concentration levels to produce toxicity symptoms in cucumbers, as also supported by the measurements of relative chlorophyll contents (Figure 2A) and by previous observations (Ramírez-Rodríguez et al., 2020b; Carmona et al., 2021; Gaiotti et al., 2021; Pérez-Álvarez et al., 2021). These data are coherent with the fact that the NP used in this experiments was essentially composed of Ca and P, and that several authors have previously used it as P fertilizers for crop plants (Liu and Lal, 2014; Marchiol et al., 2019). Indeed, P is an essential macronutrient for plants (Hopkins and Hansen, 2019); however, using the currently available fertilization approaches, only a minor part (not more than 10%) is actually used by plants, mainly because of low P use efficiency (PUE) (Kopittke et al., 2019). Such scenario is additionally worsened by the accumulation of heavy metals in agricultural soils, for instance copper (Cesco et al., 2021), which can interfere with the P uptake mechanisms, thus contributing to further limitation in the ability of plants to acquire the nutrient (Feil et al., 2020). In this context, the application of nanofertilizers characterized by the slow release of P (Liu and Lal, 2014; Carmona et al., 2020; Ramírez-Rodríguez et al., 2020a), alongside urea, could possibly allow for the increase of PUE in crops. On the other hand, the enhanced concentration of Ca in plant tissues can also represent a benefit brought about by NP-based nonofertilizers. Calcium is an essential element for both plants and animals, thereby playing different roles, from both structural and biochemical (i.e., signaling) points of view (Sharma et al., 2017). Being the major staple crops, poor sources of Ca, the application of fertilization strategies that lead to Ca fortification in the edible part of plants might indeed increase their nutritional value. In addition, recent evidence has also pointed out that an enhanced concentration of Ca in the plant tissue could be related to higher resistance against the infection of fungal pathogens (Cesco et al., 2020), since it plays a crucial role in the stabilization and strengthening of the cell wall (Elmer and Datnoff, 2014). On the other hand, the urea-treated plants showed a prominent accumulation of Mg, S, and Zn (Figure 5), which is consistent with data recently obtained from hydroponically-grown maize plants fully fertilized with bulk urea (Buoso et al., 2021a,b). Even though literature concerning the synergistic or antagonistic relationship between either urea or NP and other mineral nutrients is still lacking, the higher Zn accumulation observed in the urea-treated plants with respect to the NP-treated ones could be ascribed to Zn/P antagonism; indeed, for several plant species, the increased uptake of P has been shown to inhibit the ability of plants to take up Zn, often causing the appearance of Zn deficiency symptoms (Reed, 1946; Cakmak and Marschner, 1987; Broadley et al., 2010; Santos et al., 2021).

## Conclusion

In conclusion, this research aimed to understand the physiological and molecular responses of urea uptake mechanism in the model plant *C. sativus* L. upon fertilization with innovative nanofertilizers based on urea-doped amorphous calcium phosphate nanoparticles (NPs). The data hereby obtained demonstrated that the slow release of urea from NPs and/or the chemical composition of NPs themselves could contribute to the up-regulation of the urea uptake system for a longer period as compared to plants treated with bulk urea. This prolonged activation was indeed mirrored by higher accumulation of N in the NP-treated plants, even when the concentration of urea conveyed was halved (i.e., NP.5×treatment), thus confirming the higher performance previously described (Carmona et al., 2021). However, the higher accumulation of N was only partially translated to a higher biomass production, at least in this experience. In addition, besides impacting N nutrition, NPs also enhanced Ca and P concentration in cucumber tissues, which might possibly affect plant growth and yield, and the nutritional value of the agricultural product. Nevertheless, future studies will be necessary to address the suitability of NP-based fertilization strategies for different crops cultivated in different soil types under open field conditions.

## Data Availability Statement

The original contributions presented in the study are included in the article/Supplementary Material, further inquiries can be directed to the corresponding author/s.

## Author Contributions

NM, FG, SC, and YP: experimental design. SF, GR, MA, and FC: experiment execution. SF, GR, MA, and YP: data collection. SF, GR, MA, FC, and YP: data analyses and visualization. SC and YP: data interpretation. SF, FG, SC, and YP: manuscript writing and critical revision. NM and YP: financial support. All authors contributed to the article and approved the submitted version.

## Conflict of Interest

The authors declare that the research was conducted in the absence of any commercial or financial relationships that could be construed as a potential conflict of interest.

## Publisher’s Note

All claims expressed in this article are solely those of the authors and do not necessarily represent those of their affiliated organizations, or those of the publisher, the editors and the reviewers. Any product that may be evaluated in this article, or claim that may be made by its manufacturer, is not guaranteed or endorsed by the publisher.

## Footnotes

1
https://phytozome-next.jgi.doe.gov/

2
www.cpiinternational.com

## Supplementary Material

Supplementary Figure 1 Spectroscopic and diffractometric analyses of U-ACP. Left: FTIR spectra of pure urea (in red), pure ACP (gray), and the U-ACP nanocomposite (black). In the latter spectrum, the starred peaks are assigned to vibrational modes of urea molecules. Right: XRPD spectra collected on of pure urea (in red), pure ACP (gray) and the U-ACP nanocomposite (black). The latter spectrum clearly shows that urea is absorbed by/occluded in ACP and does not appear as a crystalline material.

Supplementary Figure 2 Schematic drawing representing the experimental plans. **(A)** Preliminary induction of cucumber plants with 1 mM Urea and determination of urea uptake rate and *CsDUR3* expression. **(B)** Induction of cucumber plants with either 1 mM Urea, NP, or NP.5×, and determination of urea uptake rate and *CsDUR3* expression. **(C)** Mid-term experiment in which cucumber plants have been either not treated (control) or treated with 1 mM Urea, NP or NP.5×for 7 days, for the following determination of growth parameters (i.e., SPAD index, root and shoot biomass, root architecture, and ionomic signature).

Supplementary Figure 3 Urea uptake in cucumber plants either not induced or induced with Urea. **(A)** High-affinity urea uptake rate in cucumber roots either not treated (not induced) or treated with 1 mM Urea. Uptake rates were determined by placing the seedlings in 200 μM^15^N-labeled urea solution for 7 min. Data are the means (± SE) of three independent biological replicates; each biological replicate was obtained by pooling five independent plants. The statistical significance within each time point has been assessed by Student’s *t*-test. **(B)** Gene expression analysis of *CsDUR3*in cucumber roots either not treated (not induced) or treated with 1 mM urea at 8HAT. *CsDUR3* expression levels were assessed by qRT-PCR; data have been normalized to two internal controls, ubiquitin elongation protein and elongation factor 1-α tubulin. The relative expression ratios were calculated using not-induced roots sampled as calibrator. The data are means (±SE), *n* = 3. The statistical significance within each time point has been assessed by Student’s *t*-test.
