# Supplementary figures and images for: Physiological and Molecular Investigation of Urea Uptake Dynamics in Cucumis sativus L. Plants Fertilized With Urea-Doped Amorphous Calcium Phosphate Nanoparticles
Source: Front Plant Sci. 2021 Dec 7;12:745581. doi: 10.3389/fpls.2021.745581 (PMC8688946; doi:10.3389/fpls.2021.745581)

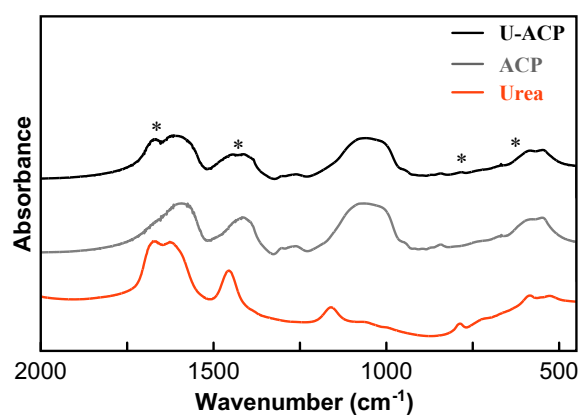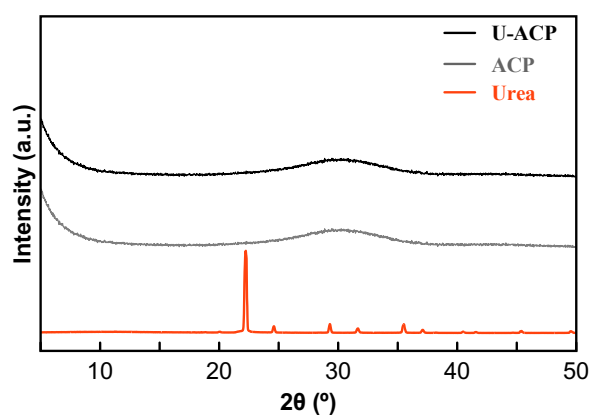

Supplement: Supplementary Figure 1 — Spectroscopic and diffractometric analyses of U-ACP. Left: FTIR spectra of pure urea (in red), pure ACP (gray), and the U-ACP nanocomposite (black). In the latter spectrum, the starred peaks are assigned to vibrational modes of urea molecules. Right: XRPD spectra collected on of pure urea (in red), pure ACP (gray) and the U-ACP nanocomposite (black). The latter spectrum clearly shows that urea is absorbed by/occluded in ACP and does not appear as a crystalline material. [file Image_1.pdf]

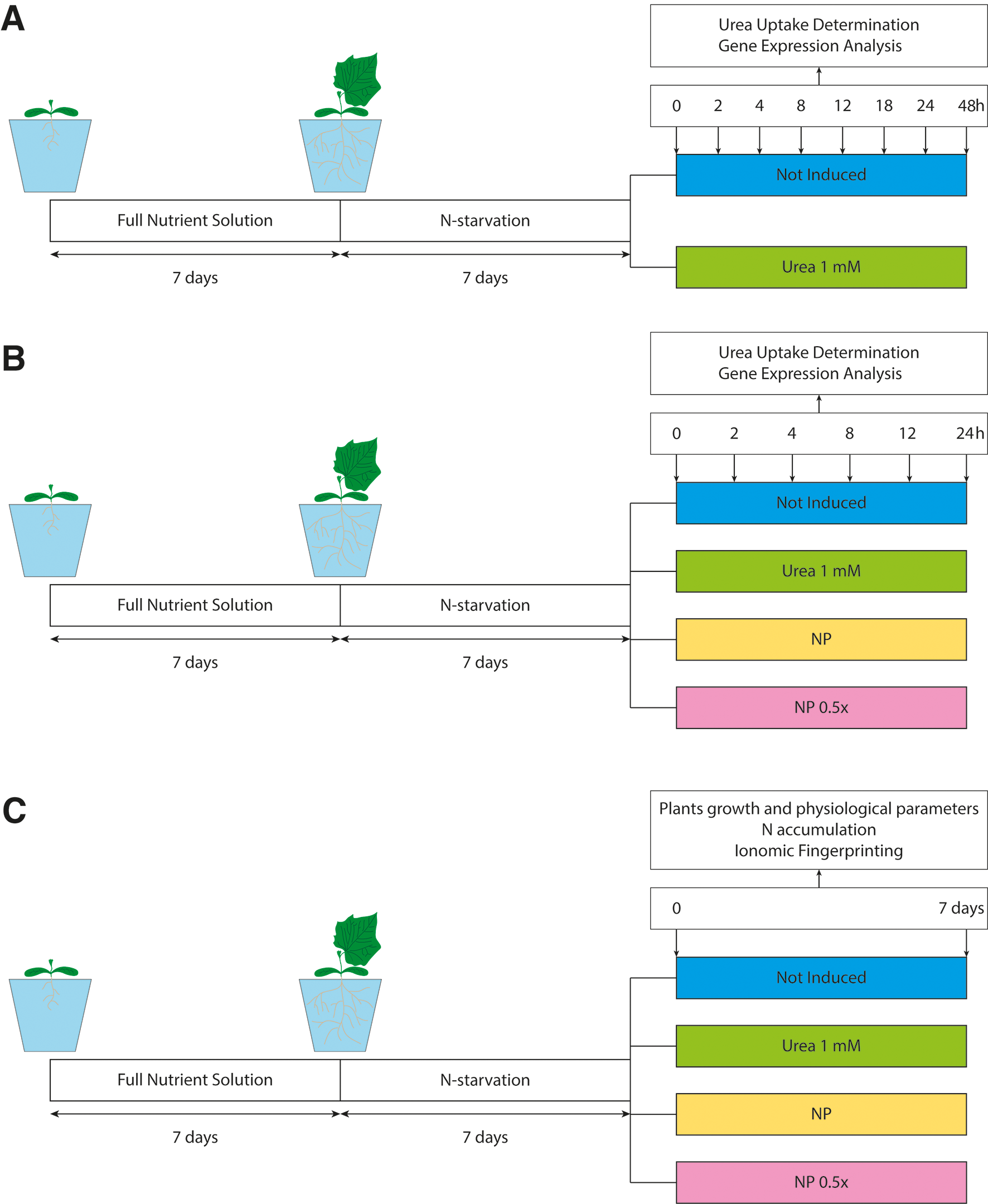

Supplement: Supplementary Figure 2 — Schematic drawing representing the experimental plans. (A) Preliminary induction of cucumber plants with 1 mM Urea and determination of urea uptake rate and CsDUR3 expression. (B) Induction of cucumber plants with either 1 mM Urea, NP, or NP.5×, and determination of urea uptake rate and CsDUR3 expression. (C) Mid-term experiment in which cucumber plants have been either not treated (control) or treated with 1 mM Urea, NP or NP.5×for 7 days, for the following determination of growth parameters (i.e., SPAD index, root and shoot biomass, root architecture, and ionomic signature). [file Image_2.tif]

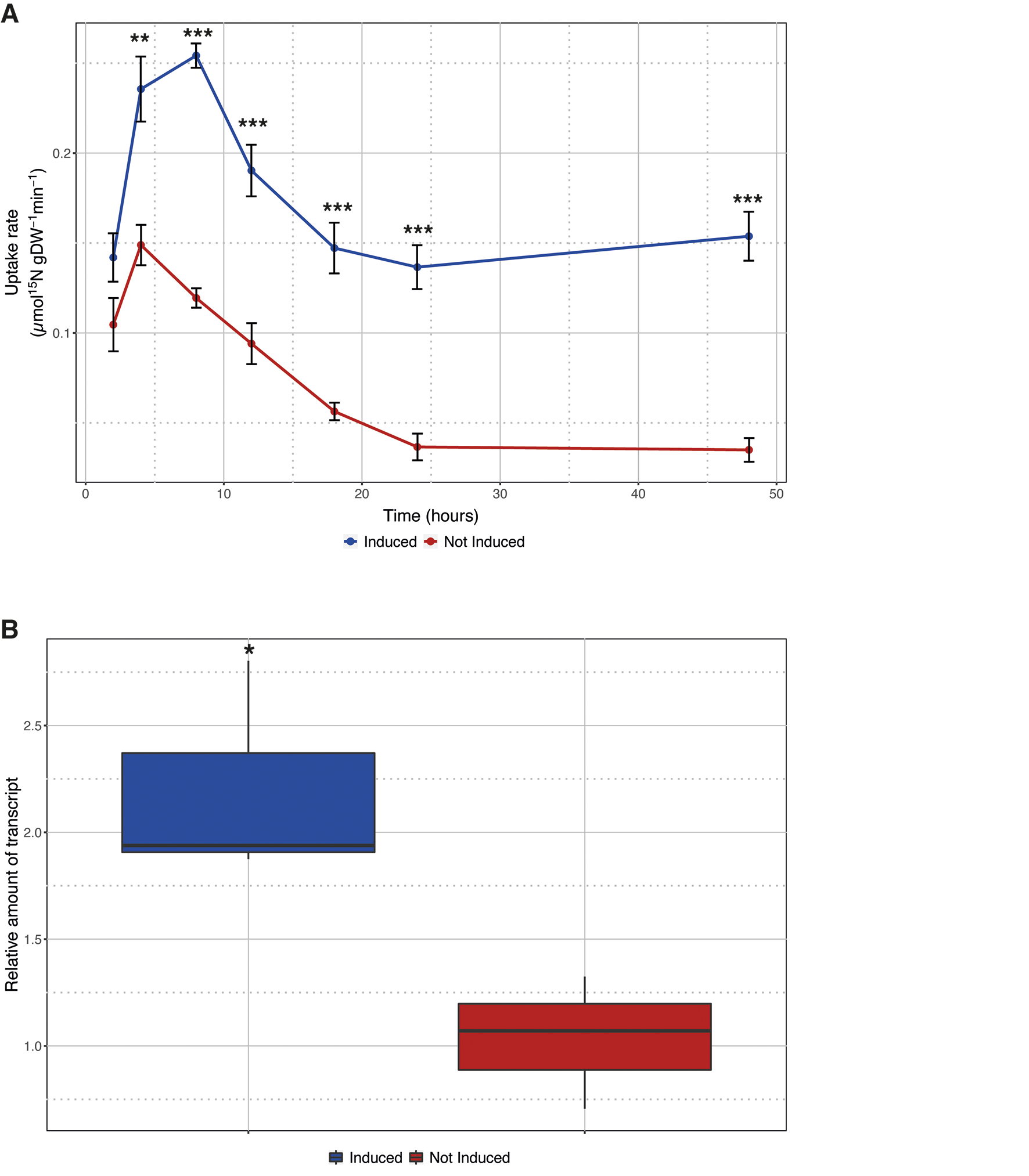

Supplement: Supplementary Figure 3 — Urea uptake in cucumber plants either not induced or induced with Urea. (A) High-affinity urea uptake rate in cucumber roots either not treated (not induced) or treated with 1 mM Urea. Uptake rates were determined by placing the seedlings in 200 μM15N-labeled urea solution for 7 min. Data are the means (± SE) of three independent biological replicates; each biological replicate was obtained by pooling five independent plants. The statistical significance within each time point has been assessed by Student’s t-test. (B) Gene expression analysis of CsDUR3in cucumber roots either not treated (not induced) or treated with 1 mM urea at 8HAT. CsDUR3 expression levels were assessed by qRT-PCR; data have been normalized to two internal controls, ubiquitin elongation protein and elongation factor 1-α tubulin. The relative expression ratios were calculated using not-induced roots sampled as calibrator. The data are means (±SE), n = 3. The statistical significance within each time point has been assessed by Student’s t-test. [file Image_3.tif]
