# Supplementary material for: Physiological and Molecular Investigation of Urea Uptake Dynamics in Cucumis sativus L. Plants Fertilized With Urea-Doped Amorphous Calcium Phosphate Nanoparticles
Source: Front Plant Sci. 2021 Dec 7;12:745581. doi: 10.3389/fpls.2021.745581 (PMC8688946; doi:10.3389/fpls.2021.745581)
Supplement: Supplementary file 4 [file Data_Sheet_1.docx]

**Supplmentary Material**

**Supplmentary Table 1.** ANOVA analysis of the data reported in Figure 1A (p<0.05).

|  | **Time after the treatments(h)** | | | | |
| --- | --- | --- | --- | --- | --- |
| **Treatment** | 2 | 4 | 8 | 12 | 24 |
| Not Induced | c | c | b | b | b |
| NP | ab | a | a | a | a |
| NP 0.5x | a | b | a | a | a |
| Urea | bc | bc | a | b | b |

**Supplementary Information 1**. Software and R packages used for the statistical analyses and data representation.

R version 3.6.1 (2019-07-05)

Platform: x86_64-apple-darwin15.6.0 (64-bit)

Running under: macOS 10.16

Matrix products: default

LAPACK: /Library/Frameworks/R.framework/Versions/3.6/Resources/lib/libRlapack.dylib

Packages:

- vegan_2.5-7
- lattice_0.20-38
- permute_0.9-5
- fmsb_0.7.0
- carData_3.0-2
- agricolae_1.3-1
- plyr_1.8.4
- ggplot2_3.2.1
